# Mitochondrial quality control at the ferroptosis-pyroptosis interface in diabetic cardiomyopathy: a narrative critical synthesis

**DOI:** 10.3389/fcvm.2026.1950950

**Published:** 2026-10-12

**Authors:** Shibo Sun, Jiarui Li, Mingxiu Sun, Xin Song, Ming Tang, Likun Du

**Affiliations:** 1 Heilongjiang University of Chinese Medicine, Harbin, China; 2 Department of Endocrinology II, First Affiliated Hospital, Heilongjiang University of Chinese Medicine, Harbin, China

**Keywords:** diabetic cardiomyopathy, ferroptosis, heart failure, metabolic inflammation, mitochondrial quality control, pyroptosis, regulated cell death

## Abstract

Diabetic cardiomyopathy is clinically heterogeneous, whereas much of its mechanistic literature relies on experimental models. This narrative critical synthesis examines whether mitochondrial quality control links diabetic stress to ferroptosis, pyroptosis, and multicellular remodeling. The evidence base is an author-selected set of 112 publications mapped from a 400-record collection, supplemented by methodological and clinical context. Original database-specific search histories were not retained in the available materials. PubMed Central full text was available for 66 corpus records; 46 were initially accessible only as abstracts. A focused full-text reassessment of selected original reports replaces the earlier corpus-wide support labels. We distinguish core quality-control processes from associated metabolic regulators and separate death markers from functional evidence of execution. The best-supported findings concern model-specific changes in mitochondrial function, lipid-peroxide-sensitive injury, and intercellular communication. They do not establish a universal mitochondrial quality-control hierarchy or same-cell ferroptosis-pyroptosis co-execution. No single study identified in the available evidence demonstrates all three transitions from markers to execution, from one death program to another, and from rodents to a corresponding human mechanism. We compare same-cell coupling with cross-cell propagation and specify experiments that would distinguish them. Human phenotype and prognosis associations support disease relevance but cannot establish myocardial death mechanisms or therapeutic mediation. The resulting framework treats mitochondrial quality control as interacting, separable processes that may alter susceptibility to particular death programs, rather than as an established master regulator.

## 1 Introduction

Diabetic cardiomyopathy (DbCM) refers to myocardial dysfunction in people with diabetes that cannot be explained solely by coronary disease, hypertension, or valvular disease. The clinical construct is heterogeneous. Early phenotypes may include myocardial steatosis, impaired energetics, abnormal strain, and diastolic dysfunction despite preserved ejection fraction. Later presentations include overt heart failure with preserved ejection fraction, mixed ventricular-vascular dysfunction, and, in a subset, reduced systolic function. Obesity, kidney disease, aging, biological sex, and vascular disease modify these trajectories and remain competing explanations in human cohorts (1–3).

No experimental model reproduces the full clinical spectrum. Type 1 diabetes models emphasize insulin deficiency and hyperglycemia, whereas type 2 diabetes models usually combine insulin resistance, obesity, and lipid oversupply. Human engineered cardiac tissue improves species relevance and can model early lipotoxic injury, but it does not reproduce chronic hemodynamic load or multimorbidity. Mechanistic claims must be triangulated across models selected for the relevant disease stage and causal question (1, 4, 5).

Regulated cell death (RCD) is defined by an execution program that can be modified by genetic or pharmacological intervention. This distinction matters because many DbCM studies infer ferroptosis or pyroptosis from stress markers, inflammatory signaling, or non-specific loss of viability. The relevant causal question is whether the proposed executor is necessary for cardiac-cell loss and whether its inhibition changes tissue remodeling. A shared upstream stressor alone cannot establish shared execution (6–9).

Existing reviews describe mitochondrial dysfunction, ferroptosis, and inflammatory death in DbCM, but their coexistence does not establish a common causal sequence. This review focuses on the inferential gap between mitochondrial perturbation and identified death execution. Its central comparison is between same-cell ferroptosis-pyroptosis coupling and propagation between different cardiac cell populations. Table 1 presents the observations that would discriminate these explanations; Table 2 maps the current cell-type-specific evidence. The contribution is a bounded critical interpretation of selected studies, not a comprehensive estimate of the field (7–11).

**Table 1:** Discriminating experiments and the three unresolved inferential transitions.

Question	Discriminating experiment	Interpretation/present boundary
Markers → execution	Measure death with a program-proximal readout and matched functional rescue	A changed marker is not equivalent to a prevented death event
One program → another	Time-resolved, reciprocal ferroptosis and gasdermin perturbation with independent death readouts	Asymmetric rescue supports direction; parallel marker changes do not
Same cell or different cells?	Locate both programs in defined cells; compare cell-restricted rescue with interruption of communication	Shared upstream stress, same-cell coupling, and cross-cell propagation remain alternatives
MQC upstream or secondary?	Restore a defined core process after injury onset; verify function/flux and subsequent death	Persistent death despite successful restoration weakens the proposed upstream link
Rodent → human mechanism	Repeat the specified perturbation and death readouts in a human-relevant model; assess patient relevance separately	A human association or engineered tissue response alone is not clinical causal validation
Current integrated evidence	No single study identified in the available material demonstrates all three transitions above	This is an evidence gap, not proof that the proposed biology is absent

**Table 2:** Cell-type-specific evidence at the ferroptosis-pyroptosis interface.

Cell type	Ferroptosis	Pyroptosis	Core-MQC dependence	Coupling/communication
Cardiomyocytes/cardiomyoblasts	SS-31 and FGF21: viability plus lipid-peroxidation rescue; PACS2 attribution incomplete (5, 13, 16)	NLRP3, CD38, and GDF11: injury/cleavage evidence; gasdermin necessity unresolved (11, 15, 17)	Dusp15-mtHsp70 supports proteostasis, not RCD execution (12)	Same-cell ferro-pyro execution not established. Vesicle-mediated fibroblast responses demonstrated, not a death relay (14, 19)
Endothelial cells	DNA-PK intervention: oxidative and injury readouts, without matched ferroptotic death rescue (18)	Not established by the reassessed endothelial report	No core-MQC-to-death mediation established in that report	Vascular improvement does not identify a donor-recipient death sequence
Macrophages	Not established in the focused audit	Inflammatory signaling is not sufficient to assign macrophage pyroptosis	S100A9-STAT3 route is nominated from an abstract; not full-text graded here (76)	Macrophage-to-cardiomyocyte communication is a candidate route; death-specific transfer unresolved
Fibroblasts	Not established by the reassessed vesicle studies	Inflammatory/profibrotic responses are not pyroptotic execution	Donor mitochondrial interventions do not establish recipient core-MQC mediation (14)	Vesicle uptake/cargo perturbation supports communication (14, 19); same-cell coupling and propagated death remain unresolved

**Table 3:** Bounded claim-level audit of seven illustrative death-focused reports.

Category/count	Operational rule	Illustrative reports	Claim retained/boundary
A: incomplete death attribution 5 of 7	The joint requirements of death measurement, program-proximal readout, and matched rescue of death are not established for the claim assessed	PACS2 (5); NLRP3 (11); CD38 (15); GDF11 (17); endothelial DNA-PK (18)	Perturbation-associated injury or susceptibility; no certified executor dependence
B: death-rescue support 2 of 7	Death/viability, lipid peroxidation, and ferrostatin-1 rescue are reported; core-MQC mediation remains unresolved	SS-31 (13); FGF21 (16)	Pharmacological support for lipid-peroxide-sensitive injury; not proof of a specific mitochondrial mediator
C: core-MQC-mediated execution 0 of 7	Category B-level death attribution plus all four core-MQC requirements in Section 2.3	None among these seven reports	No field-wide absence claim; the denominator is illustrative and selected
Beyond this audit	Same-cell coupling, cross-program direction, and human mechanism are separate claims	See Tables 1, 2	No aggregate score, validated risk-of-bias grade, or inter-rater statistic

## 2 Evidence scope and a testable modular framework

The review separates the provenance of its literature collection from the strength of individual mechanistic claims. The former can be documented without implying that the latter has been established.

### 2.1 Review approach, evidence base, and claim boundaries

This article is a narrative critical synthesis of author-selected literature, not a systematic review or meta-analysis. The available source workbook contains 400 candidate records. A second workbook organizes 112 unique publications into 250 section-reference assignments; assignments are repeated uses of publications, not additional studies. Selection was organized around diabetic cardiac phenotypes, mitochondrial mechanisms, regulated cell death, and clinical or methodological context. This describes the documented editorial selection rationale, not prospectively applied eligibility criteria. Two records flagged as retracted in the source collection were excluded from the selected set (Figure 1). The collection principally covers 2021–2026, with older definitions and methods cited separately. Database-specific original search strings, last search dates, duplicate-screening decisions, and a complete screening log cannot be reconstructed from these workbooks. No prospective protocol registration or independent duplicate screening is claimed.

**Figure 1:**
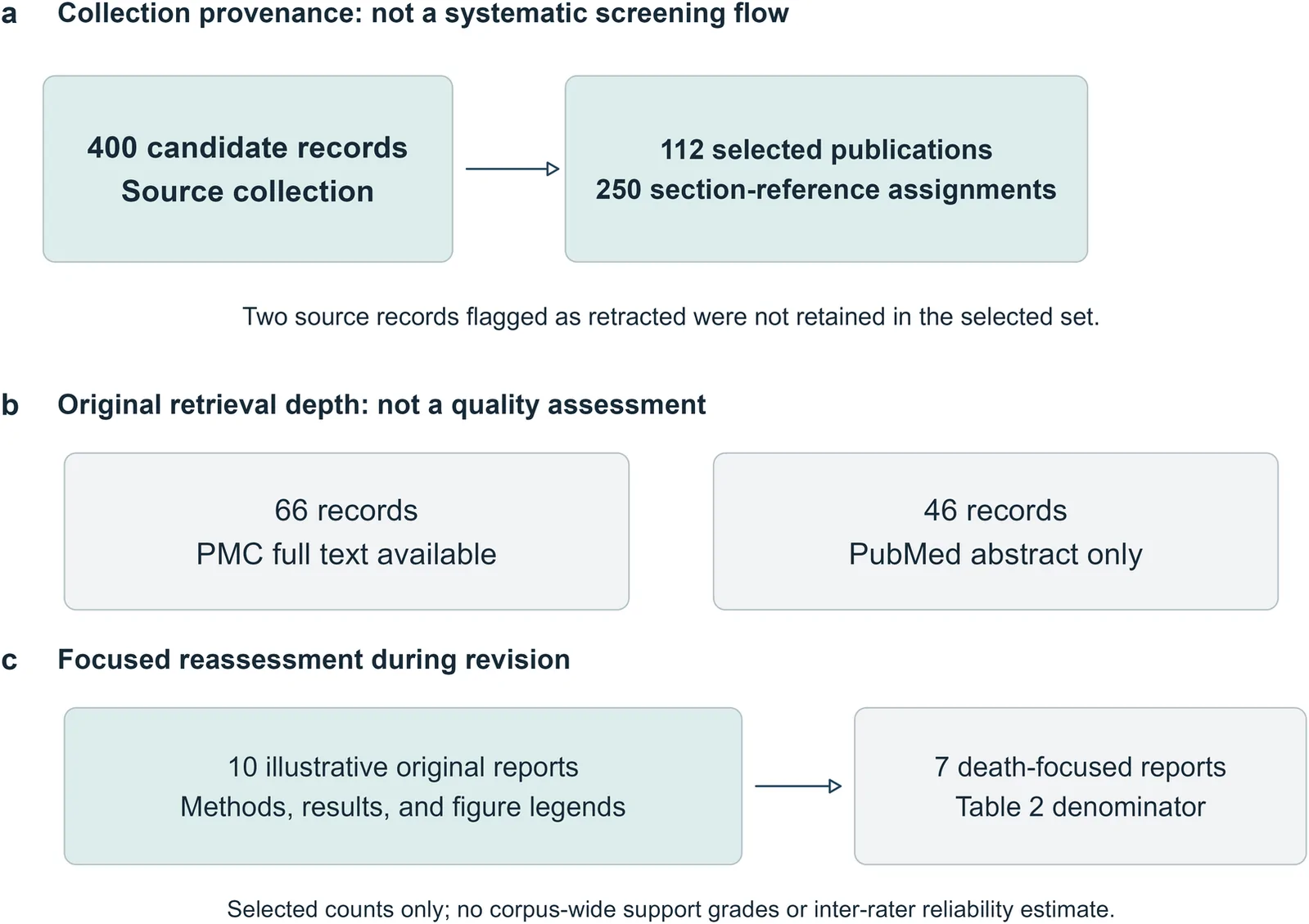
Provenance and limits of the author-selected evidence base. **(a)** A 400-record source collection was mapped to 112 selected publications and 250 section-reference assignments. Two source records flagged as retracted were not retained in the selected set. This is a provenance diagram, not a systematic-review screening flowchart. **(b)** Archived retrieval depth: PMC full text was available for 66 records and only PubMed abstracts for 46. These counts do not measure study quality or completed full-text appraisal. **(c)** Ten illustrative original reports underwent focused full-text reassessment during revision; seven death-focused reports supply the denominator for Table 3. No independent duplicate appraisal or inter-rater reliability estimate is claimed. All counts refer to this collection or the explicitly defined subset, not to field-wide prevalence.

All 112 selected records have PubMed identifiers. Archived PubMed Central (PMC) full-text material was available for 66 records, whereas 46 were initially accessible only as PubMed abstracts. Availability is not equivalent to a completed methodological appraisal. During revision, the full-text methods, results, and figure legends of ten illustrative original reports were reassessed for the particular claims discussed below: PACS2, NLRP3, Dusp15, SS-31, IL-37, CD38, FGF21, GDF11, endothelial DNA-PK, and cardiomyocyte-derived vesicles. These reports were selected because they directly inform disputed death-attribution, proteostasis, or communication claims, not as an exhaustive or representative sample. The seven death-focused reports form the bounded denominator in Table 3; the other three provide mechanistic context. Supplementary Table S1 records the original retrieval depth for every corpus publication, and Supplementary Table S2 specifies the focused reassessment and its remaining gaps. We did not calculate a formal risk-of-bias score, pooled effect, or inter-rater agreement statistic (5, 11–19).

A revision-stage PubMed metadata check on September 10, 2026, used the submitted reference list's 121 PMIDs with the NCBI EFetch service (db = pubmed; retmode = xml). Publication types and linked correction notices were checked for retraction flags; none of the 121 records was flagged as a retracted publication or linked to a retraction notice at that check. This was an identifier-based status check, not a new literature search. Abstract-only records are used for reported scope or contextual findings, not to establish unreported genetic necessity, rescue specificity, flux, or study quality. Corpus-wide preliminary support tiers have been removed. The criteria below are interpretive rules for explicit claims, not a validated grading instrument; counts describe only the stated denominator.

### 2.2 Core MQC processes and associated regulators

We use MQC narrowly for processes that maintain, repair, remove, or replace mitochondrial components: mitochondrial dynamics, selective mitochondrial clearance, biogenesis and replacement, and intramitochondrial proteostasis. These processes interact but remain experimentally separable. Morphology, protein abundance, and respiration are informative readouts, but no one readout establishes successful quality control. In particular, PINK1, Parkin, or LC3 abundance cannot distinguish increased clearance from blocked turnover (20–22).

Cristae organization and cardiolipin remodeling describe structural maintenance that may interact with core MQC. Organelle contacts, calcium handling, iron availability, substrate metabolism, NAD + balance, and mtDNA release are treated here as associated regulators or consequences, not additional MQC modules by definition. Their inclusion requires a specified connection to mitochondrial maintenance or death susceptibility. This boundary prevents MQC from becoming a synonym for all mitochondrial biology. Tissue inflammation and fibrosis are downstream phenotypes, not components of MQC (20, 23, 24).

An increase in fission may segregate damaged segments or contribute to persistent fragmentation. Increased mitophagy may preserve quality or deplete mitochondrial reserve if replacement is inadequate. The direction of the response therefore depends on turnover, energetic recovery, and timing, rather than on whether a marker rises or falls. The working model is that separable MQC processes alter susceptibility to particular injuries under defined conditions; it does not assume that all processes act in the same direction (25–27).

A proposed commitment threshold remains a hypothesis. Membrane-potential loss, lipid peroxidation, ATP or NAD + depletion, mtDNA release, and impaired clearance can accompany several forms of injury. Their coexistence does not identify an irreversible state or a common death executor. A relevant experiment would restore a defined MQC process after injury begins and test whether mitochondrial function and survival remain recoverable (25, 26, 28).

### 2.3 Claim-specific criteria and competing explanations

We separate three questions: is a death program functionally implicated; is a core MQC process upstream of that death; and does the mechanism extend across cells or into human disease? For the seven illustrative death-focused studies in Table 3, category A denotes failure to establish the joint requirement of measured death, a program-proximal readout, and mechanism-matched rescue of death. Category B requires all three, but does not establish core-MQC mediation. Category C additionally requires perturbation of a defined core MQC process, a corresponding functional or flux readout, temporal precedence, and a rescue or epistasis experiment linking that process to the identified death program. Categories are assigned to the claim assessed, not to the overall quality of a paper. Pharmacological evidence can support category B without establishing molecular specificity (6, 29, 30) (Figure 2).

**Figure 2:**
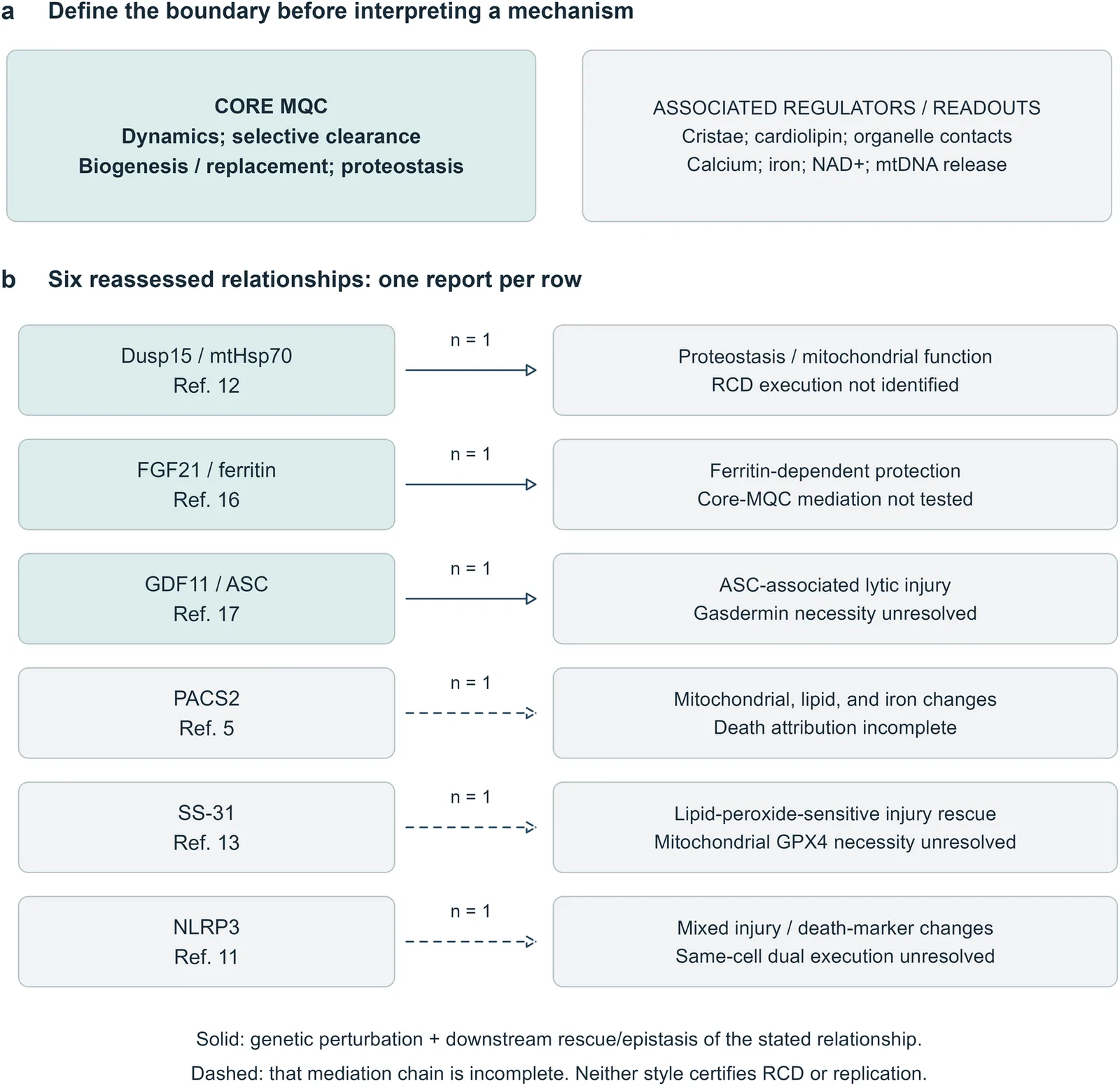
Study-specific relationships and their inferential limits. **(a)** Defines the boundary before interpreting a mechanism: core MQC processes (dynamics; selective clearance; biogenesis/replacement; proteostasis) are distinguished from associated regulators/readouts (Cristae; cardiolipin; organelle contacts; calcium; iron; NAD+; mtDNA release). **(b)** Six reassessed relationships, one report per row, with *n* = 1 per relationship. Solid connectors indicate a genetic perturbation plus a downstream rescue or epistasis test of the stated relationship; dashed connectors indicate an incomplete genetic-rescue chain for the stated mediation claim. [Explanations for PACS2, SS-31, NLRP3 and the abbreviation list remain unchanged.]

Among these seven selected reports, five were placed in category A, two in category B, and none in category C (Table 3; Supplementary Table S2). The counts are an audit of illustrative claims, not an estimate of the frequency or quality of evidence across the 112-record collection. For pyroptosis, gasdermin cleavage with lytic injury is distinguished from demonstrated gasdermin dependence. For ferroptosis, altered iron, GPX4 abundance, or oxidative products must be distinguished from lipid-peroxide-sensitive death. Evidence for molecular mediation, cross-program coupling, and human relevance is recorded separately rather than added into a numerical quality score.

The modular model has alternatives: mitochondrial abnormalities may follow death commitment, reflect nonspecific metabolic injury, or arise in cells different from those displaying death markers. It would be weakened if a well-verified restoration of the implicated MQC process failed to alter the identified death program, or if death consistently preceded the proposed MQC defect. Same-cell coupling requires evidence beyond a shared upstream perturbation. The minimum measurements are consolidated in Table 4, and the discriminating experiments in Table 1.

**Table 4:** Minimum evidence for MQC and regulated cell-death inference.

Claim	Required evidence	Insufficient alone
Core MQC function	Defined dynamics, clearance, replacement, or proteostasis process; functional or turnover readout matched to that process	Static PINK1, Parkin, LC3, morphology, or respiration
Ferroptotic death	Death/viability measurement, lipid-peroxidation readout, and mechanism-matched rescue of death; orthogonal perturbation strengthens attribution	Iron, MDA, 4-HNE, or GPX4 abundance
Pyroptotic death	Gasdermin-related execution and lytic injury, with gasdermin-dependent rescue to establish executor necessity	NLRP3, cytokines, or cleavage bands without functional dependence
MQC-mediated death	Death attribution plus defined core-MQC perturbation, matched functional readout, temporal precedence, and rescue/epistasis	Parallel improvement of mitochondrial and injury markers
Same-cell coupling	Both programs localized to the same cell; timed, separate perturbations establish each contribution and direction	Bulk-tissue mixed markers or one upstream inhibitor
Cross-cell propagation	Identified sender, transfer route, recipient, and route-specific interruption; death attribution in relevant compartments	Cytokine or vesicle effects on fibrosis alone
Human mechanism	Perturbation-linked mechanism in a human-relevant system; human disease and efficacy tested separately	Human-cell origin, biomarker association, or clinical benefit without mediation

## 3 Diabetic stress and failure of mitochondrial quality control

Diabetic stress must be linked to failed mitochondrial repair, segregation, removal, or replacement before MQC can be placed upstream of death. We organize the evidence from substrate overload to checkpoint failure, and identify measurements that cannot establish causality.

### 3.1 Substrate overload and loss of metabolic flexibility

Chronic hyperglycemia increases reducing pressure on the respiratory chain, promotes reactive metabolite formation and can uncouple glucose availability from efficient glucose oxidation. The resulting mismatch raises mitochondrial electron leak while diverting intermediates into glycation, polyol and hexosamine pathways. Glucose excess alone, however, does not reproduce the full human phenotype. Its impact depends on insulin signaling, lipid delivery, workload and antioxidant reserve, which may explain why identical glucose concentrations can produce different mitochondrial outcomes across models (31–33).

The diabetic heart increasingly relies on fatty-acid oxidation while receiving an oversupply of circulating fatty acids. Although this adaptation maintains ATP production, it increases oxygen cost and generates lipid intermediates that alter membranes, signaling and redox balance. Ceramides, acylcarnitines and peroxidizable phospholipids can therefore connect lipotoxicity to both MQC stress and ferroptotic susceptibility. Pathogenicity appears to depend less on fatty-acid oxidation alone than on persistent substrate inflexibility combined with incomplete oxidation and lipid remodeling (4, 34, 35).

Insulin resistance reduces metabolic flexibility and weakens the heart's ability to match substrate use to workload. Mitochondria then operate under chronic fatty-acid pressure while glucose oxidation remains constrained, increasing the probability that transient stress becomes sustained damage. Because insulin resistance is also linked to obesity, vascular dysfunction and systemic inflammation, analyses must separate myocardial mechanisms from whole-body confounding. Tissue-specific perturbations and paired metabolic flux measurements are especially important for establishing a direct cardiac pathway (36–38).

### 3.2 Redox stress, glycation, and calcium overload

Advanced glycation and oxidant production reinforce each other through receptor signaling, protein modification and impaired antioxidant defense. At the same time, altered endoplasmic-reticulum and sarcoplasmic-reticulum calcium release can overload mitochondria, stimulate ROS and promote permeability transition. Calcium is therefore positioned between upstream metabolism and downstream death commitment. Strong evidence would show that correcting mitochondrial calcium handling improves function without merely suppressing a general stress response (27, 39, 40).

Mitochondrial ROS can act as a signal, a damaging agent or an amplifier of RCD. Transient ROS may induce adaptive antioxidant and mitophagic responses, whereas sustained production oxidizes proteins, lipids and mtDNA and can overwhelm thiol-based defense. This mechanistic threshold depends on location, duration and buffering capacity. Global fluorescent probes and end-stage oxidative products cannot establish that mitochondrial ROS is the initiating cause of death (33, 41, 42).

Mitochondrial calcium overload links excitation-contraction coupling to energetic injury, but calcium signaling does not by itself identify a death program. Moderate uptake can support ATP production, whereas sustained overload can accompany permeability transition, oxidant stress, and loss of membrane potential. The relevant distinction is between an upstream regulator of susceptibility and a late consequence of cellular injury. The same death-attribution criteria used elsewhere apply here (27, 39).

### 3.3 Mitochondrial dynamics, mitophagy, and biogenesis

Fusion and fission determine whether mitochondrial damage is diluted, segregated or propagated. DRP1-dependent fission can facilitate selective removal, whereas uncontrolled fragmentation may impair cristae and electrical connectivity. MFN1/2 and OPA1 support fusion and inner-membrane architecture, but excessive fusion can also retain damaged material. The most informative designs combine morphology with respiratory function, fission or fusion flux, and downstream rescue rather than relying on protein abundance alone (43–45).

PINK1-Parkin, BNIP3/NIX and FUNDC1 represent overlapping routes to selective mitochondrial clearance. Their activity depends on mitochondrial depolarization, receptor state, autophagosome formation and lysosomal completion. LC3-positive structures may accumulate because of increased initiation or blocked degradation, so lysosomal flux must also be measured. In DbCM, mitophagy should be interpreted together with mitochondrial mass, biogenesis and ATP reserve to determine whether clearance restores quality or creates energetic insufficiency (46–48).

Biogenesis supplies the replacement component of MQC. PGC-1alpha coordinates transcriptional programs involving NRF1/2 and TFAM, while mitochondrial translation determines whether new respiratory-chain capacity is functional. Increased mitochondrial number without coordinated nuclear and mitochondrial gene expression may not improve quality. Therapy should aim for balanced turnover: removal of damaged organelles must be coupled to generation of mitochondria capable of sustaining cardiac workload (49–51).

### 3.4 Cristae, cardiolipin, proteostasis, and organelle contacts

Proteostasis provides a more specific MQC example than mitochondrial morphology alone. In diabetic mice and cardiomyocyte experiments, Dusp15 was linked to mtHsp70 stability and phosphorylation at T116. Cardiomyocyte-specific Dusp15 deletion worsened mitochondrial and cardiac phenotypes, while mtHsp70 phosphosite manipulations tested the proposed downstream relationship. However, the Dusp15 overexpression and mtHsp70 knock-in models were not uniformly cardiomyocyte-restricted, and pharmacological suppression of the mitochondrial unfolded-protein response was not a selective, toxicity-free test. These data support a proteostasis mechanism in the tested models; they did not establish ferroptotic or pyroptotic execution. Thus, mitochondrial chaperone regulation cannot be presented as evidence of the ferroptosis-pyroptosis interface without a separate death-specific experiment (12).

Cristae and cardiolipin require similarly bounded interpretation. Cristae disruption can impair respiratory organization, and cardiolipin remodeling can alter the membrane environment in which oxidation occurs. Yet structural preservation after a protective intervention does not show that cristae repair or a particular cardiolipin species mediates survival. In the SS-31 study, viability, mitochondrial lipid peroxidation, and GPX4-related readouts improved together, but mitochondrial GPX4 necessity was not isolated genetically. This is evidence of pharmacological protection, not a resolved cardiolipin-to-ferroptosis mechanism. A stronger test would combine species-resolved lipid measurements or defined cristae perturbation with matched death rescue. The present evidence is insufficient to place cristae or cardiolipin remodeling upstream of same-cell ferroptosis-pyroptosis coupling (13, 52, 53).

Mitochondria-associated membranes are contact regions with the endoplasmic reticulum, not an independent death pathway. They can influence calcium exchange, lipid handling, and mitochondrial positioning. PACS2 perturbation was associated with changes in CPT1A, mitochondrial morphology, iron, and lipid oxidation in diabetic models, but these changes do not establish selective mitochondrial turnover or lipid-peroxide-dependent death. Advanced glycation end products (AGEs), discussed in Section 3.2, are upstream metabolic stressors; the mitochondrial unfolded-protein response (UPRmt) is a proteostatic response. Neither should be treated as interchangeable with core MQC or RCD execution (5, 54, 55).

### 3.5 Convergent checkpoints: iron, mtDNA, and energetic failure

Mitochondrial iron contributes to respiratory and iron-sulfur proteins, while an expanded labile iron pool can increase lipid oxidation. These roles make iron handling relevant to ferroptotic susceptibility, but iron accumulation is not itself proof of ferroptosis. FGF21 experiments linked protection to ferritin stabilization: ferritin perturbation modified viability and lipid-peroxidation responses, and ferrostatin-1 rescued injury. This supports an iron-handling mechanism in those models. Ferritin turnover, however, is not equivalent to mitophagic flux, and the study does not establish core-MQC-dependent death or a pyroptotic component (5, 13, 16, 56).

Damaged mitochondria release mtDNA when membrane integrity, nucleoid maintenance or selective clearance fails. Cytosolic or vesicular mtDNA can activate cGAS-STING, TLR9 and inflammasome-linked pathways, and convert organelle damage into inflammatory signaling. The direction of this sequence remains unresolved: mtDNA release may follow mitophagy failure, or intense innate immune signaling may further suppress MQC. Time-resolved localization and nuclease-sensitive rescue are required to distinguish these possibilities (14, 25, 57).

ATP and NAD + depletion can compromise repair, mitochondrial turnover, and cellular survival. They can also occur after commitment to death. CD38 deletion or knockdown was associated with improved mitochondrial and injury readouts and reduced pyroptosis-related proteins, but the available experiments do not isolate gasdermin-dependent death or establish that a core MQC process mediates protection. These metabolic measurements should therefore be interpreted as context-dependent regulators or consequences, not as shared executors (15, 34, 58).

## 4 From MQC failure to regulated cell-death execution

The central issue is not whether mitochondrial abnormalities accompany ferroptosis- or pyroptosis-related markers. It is whether the experiments identify death, its executor or protective machinery, and the direction of its relationship to a defined mitochondrial process.

### 4.1 Identifying ferroptotic execution in the diabetic heart

Ferroptosis is lipid-peroxide-dependent regulated cell death. Iron accumulation, malondialdehyde, 4-hydroxynonenal, reduced GPX4, or altered mitochondrial morphology are insufficient individually or in combination without evidence of death and mechanism-matched rescue. Mitochondria may act as susceptibility regulators, metabolic amplifiers, secondary targets of injury, or—in a demonstrated context—necessary contributors to execution. These roles are not interchangeable, and mitochondrial dysfunction should not be described as universally required for ferroptosis (6, 29).

GPX4 and parallel lipid-peroxide defense systems constrain ferroptotic susceptibility, but altered abundance does not establish that a particular defense mediates protection. The PACS2 report links mitochondrial and lipid/iron readouts to PACS2 perturbation and includes pharmacological modulation; its reported ferrostatin-1 effects on iron and lipid signals do not, by themselves, establish rescue of cell death. The inference is therefore narrower than a causal PACS2-to-ferroptosis pathway (5, 59, 60).

Iron handling shapes ferroptotic susceptibility through uptake, storage, ferritin turnover, mitochondrial use and export. Ferritinophagy can enlarge the labile iron pool, while mitochondrial iron-sulfur and haem pathways influence both respiratory function and redox chemistry. Because systemic diabetes can alter iron indices independently of myocardial ferroptosis, cardiac compartment measurements are essential. The causal question is whether iron redistribution precedes lipid-peroxide-dependent death and whether correcting it produces selective rescue (16, 60, 61).

Ferroptosis sensitivity depends on membrane lipid composition as much as on total lipid abundance. ACSL4 and LPCAT3 promote incorporation of peroxidizable polyunsaturated fatty acids into phospholipids, whereas mitochondrial beta-oxidation and lipid trafficking influence the available substrate pool. DbCM-related lipotoxicity may prime ferroptosis without being equivalent to it. Lipidomic identification of oxidized phospholipid species is more informative than bulk MDA or 4-HNE measurements (5, 13, 56).

### 4.2 Directionality between MQC and ferroptotic susceptibility

Autophagy poses a specific interpretive problem. Ferritinophagy or lipophagy can increase ferroptotic susceptibility, whereas mitophagy, lysophagy and other selective pathways may protect against damage. Autophagy activation cannot be classified as uniformly beneficial or harmful. Claims of autophagy-dependent ferroptosis require evidence that the relevant cargo-receptor pathway is necessary for ferroptotic execution, rather than merely associated with both processes (30).

The SS-31 and FGF21 reports provide more direct functional evidence than marker shifts alone because protection includes viability rescue by ferrostatin-1 alongside lipid-peroxidation measurements. Their mechanistic limits differ. SS-31 does not establish that mitochondrial GPX4 is necessary for protection; FGF21-ferritin epistasis supports an iron-handling route but does not test a core MQC process. These two reports meet category B for the assessed death claim, not category C for core-MQC mediation. Neither tests same-cell ferroptosis-pyroptosis co-execution (13, 16).

Reviews of diabetic ferroptosis remain useful for locating candidate mechanisms, but they are not independent experimental replications. The focused audit in Table 3 therefore counts original reports rather than reviews or repeated citations. Broader prevalence claims would require a reproducible search and full-text appraisal of a defined eligible set, which the present collection does not provide (59, 62, 63).

### 4.3 Inflammasome signaling is not synonymous with pyroptotic death

Inflammasome assembly, caspase-1 activation and IL-1beta maturation can occur without terminal lytic death. Conversely, gasdermin pores alter ion flux and membrane permeability before complete rupture. Recent structural work also distinguishes gasdermin-mediated pore formation from NINJ1-mediated terminal membrane disintegration. DbCM studies should report these stages separately instead of treating one cleavage band as definitive pyroptosis (6, 64).

NLRP3 activation can support cytokine processing without demonstrating gasdermin-dependent death. In the reassessed NLRP3 report, inhibition or knockdown improved injury-related readouts and altered both gasdermin/caspase markers and GPX4/xCT. Rotenone counteracted protection, but its broad mitochondrial toxicity is not a selective test of a ferroptosis-pyroptosis link. The study does not establish independent ferroptotic death rescue, gasdermin necessity, or dual execution within an identified single cell (11, 17, 26, 65).

The GDF11-ASC study moves beyond inflammasome abundance by combining propidium-iodide-positive injury and gasdermin cleavage with ASC knockdown and overexpression experiments. It supports an ASC-associated injury mechanism in the tested cardiomyoblast model. It does not isolate gasdermin dependence, and it does not test ferroptotic execution. Similarly, reduced gasdermin cleavage after CD38 manipulation cannot determine how much of the functional protection is mediated by pyroptosis. The distinction between pathway engagement and executor dependence is retained even when the intervention is genetic (15, 17, 66, 67).

The cGAS-STING pathway offers a direct route from misplaced mtDNA to innate immune transcription. In DbCM models, lipotoxic or oxidative injury can increase cytosolic mtDNA and activate STING-associated inflammatory programs. Its relationship to NLRP3 may be upstream, parallel or reciprocal depending on cell type and timing. Genetic deletion, mtDNA depletion or targeted nuclease approaches are required to show that mitochondrial DNA, rather than nuclear DNA or secondary necrosis, initiates the response (14, 25, 57).

Mitochondrial oxidant stress, calcium disturbance, and impaired dynamics are plausible contributors to inflammasome susceptibility. Their relationship to lytic execution remains a separate question. A shared change in mitochondrial function and inflammatory markers does not specify whether mitochondria act before inflammasome activation, after it, or through a parallel injury pathway (26, 27, 65).

### 4.4 Same-cell coupling versus cross-cell propagation

The same-cell model proposes that one identified cell undergoes two functionally implicated death programs, either concurrently or in a defined sequence. The cross-cell model instead proposes that injury in one lineage changes the death susceptibility or inflammatory state of another. Bulk myocardium and mixed cultures cannot reliably distinguish these explanations. Cell-type evidence is summarized in Table 2; neither model should be inferred from correlated pathway markers alone (11, 66, 68, 69).

Inflammatory signaling may amplify lipid oxidation, while products released from oxidatively injured cells may alter inflammasome responsiveness in neighboring cells. These are plausible routes of coupling, not established reciprocal execution pathways in DbCM. Cytokine suppression can reduce tissue injury without preventing a donor cell's death, and protection of a recipient cell can occur without correcting the original mitochondrial defect (65, 70, 71).

Three alternatives remain distinguishable: parallel susceptibility to independently executed programs, a directional sequence in which one program is upstream of another, and communication between different cells. The strongest current observations establish selected components of these alternatives, not the complete sequence. In particular, direct vesicle-transfer experiments support intercellular communication, but do not establish that the sender and receiver execute ferroptosis and pyroptosis, respectively (10, 11, 14, 19).

Table 1 specifies the decisive tests. A same-cell claim requires time-resolved localization of both programs and separate, nonredundant rescue of their contribution to death. A propagation claim requires a defined sender, transferred signal, recipient response, and interruption of the route. Blocking a cytokine or extracellular-vesicle pathway without identifying death in both compartments cannot establish cross-cell propagation of death itself.

The same standards apply to adjacent RCD programs. Apoptosis-related cleavage or TUNEL positivity should not be treated as a complete execution mechanism, and necroptosis-related RIPK1/RIPK3 changes require evidence for the downstream MLKL-dependent process and rescue. These categories may coexist with mitochondrial injury without explaining the ferroptosis-pyroptosis interface (6, 15, 72, 73).

Cuproptosis, PARthanatos, and PANoptosis remain emerging categories in this DbCM literature. Copper-related expression signatures do not establish copper-dependent death; PARP activity alone does not establish PARthanatos; and mixed apoptosis, pyroptosis, and necroptosis markers do not establish an integrated PANoptotic mechanism. Each claim requires its own execution-linked perturbation and rescue evidence. They are retained as boundaries of the present framework, not as additional validated outputs of a universal MQC pathway (28, 74, 75).

Thus, the ferroptosis-pyroptosis interface is an unresolved causal question rather than a demonstrated integrated death program. Figure 3 summarizes the competing explanations and the current evidential judgment. A model of interacting, separable susceptibility processes is compatible with the data without requiring same-cell co-execution (7, 10, 11, 23).

**Figure 3:**
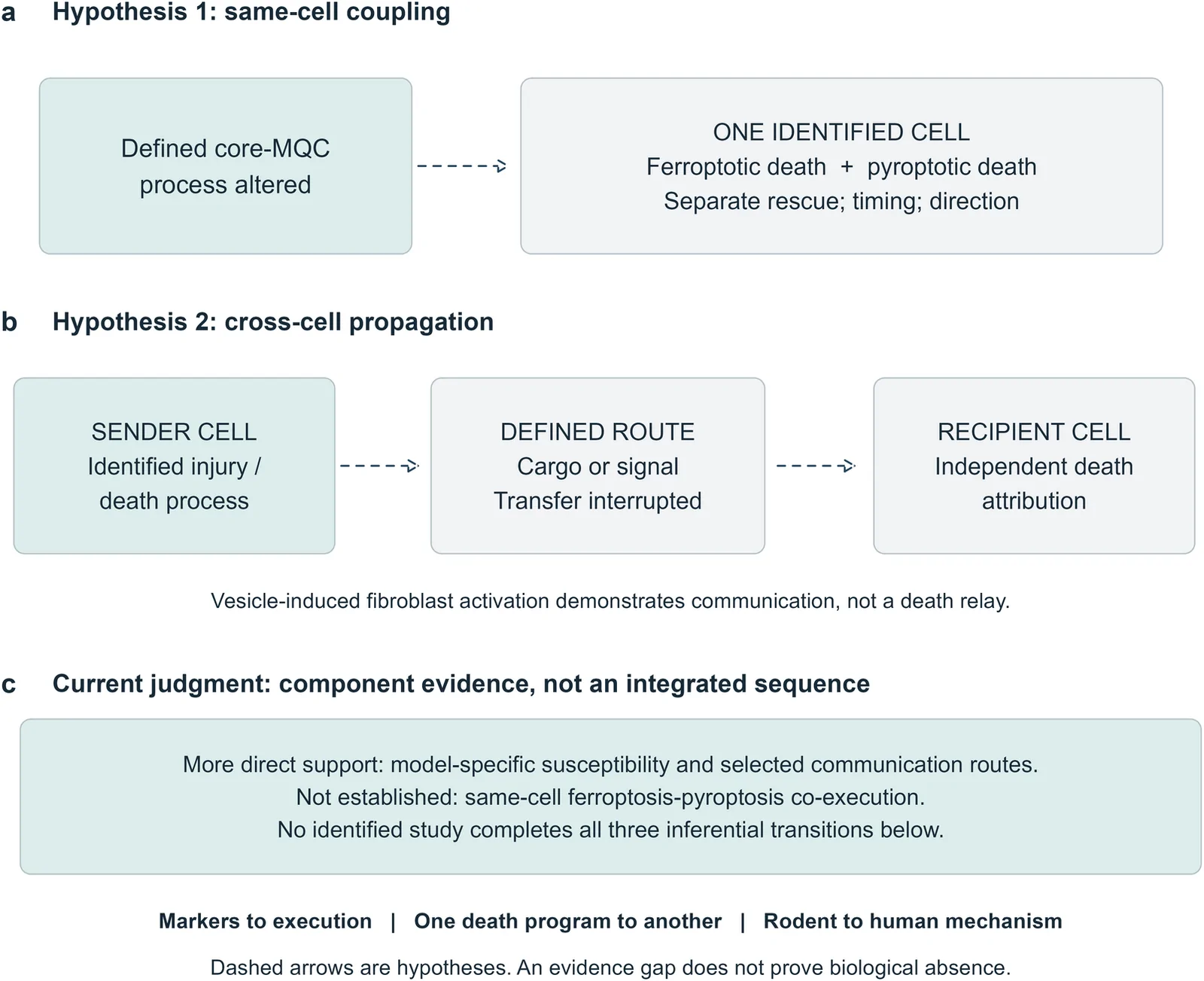
Competing explanations for mixed ferroptosis-pyroptosis signals. **(a)** Same-cell coupling requires both death processes to be localized and functionally implicated within the same cell, with temporal and reciprocal rescue experiments. **(b)** Cross-cell propagation requires donor and recipient identities, a defined transfer route, and death-specific evidence in each relevant compartment. Vesicle-induced fibroblast activation is evidence of communication, not automatically of propagated ferroptosis or pyroptosis. **(c)** Current judgment: module-specific changes in susceptibility and selected communication routes are supported more directly than integrated co-execution. The available evidence does not identify one study that demonstrates all three transitions—markers to execution, one death program to another, and rodent findings to a corresponding human mechanism. This is an evidence gap within the assessed material, not proof that such biology is absent. Dashed arrows indicate hypotheses; no temporal sequence is asserted as established.

## 5 Multicellular and spatial propagation

Organ-level DbCM cannot be explained by cardiomyocyte death alone. Mitochondrial danger signals have clinical relevance when they alter endothelial, immune, and fibroblast states and propagate focal stress into diffuse remodeling.

### 5.1 The cardiomyocyte-endothelial axis

Cardiomyocytes have high energetic demands, but whole-heart measurements cannot assign a mixed death signature to them. The reassessed studies include cardiomyoblast lines, primary cardiomyocytes, and bulk myocardium, which differ in maturity, metabolism, and cellular composition. Single-cell maps can identify candidate populations and interactions; they cannot establish lethal execution from transcription alone (11, 19, 23, 25).

Endothelial injury may alter perfusion and the cardiac microenvironment. In the reassessed DNA-PK study, pharmacological inhibition improved vascular and cardiac readouts together with oxidative products and ferroptosis-associated proteins. Without lipid-peroxide-dependent death rescue or a core-MQC perturbation linked to death, these findings support endothelial injury and susceptibility rather than a resolved MQC-ferroptosis pathway. This distinction prevents a vascular improvement from being reclassified as cardiomyocyte death rescue (18, 45, 77).

### 5.2 Macrophage states, fibroblast activation, and matrix feedback

Macrophages can transmit inflammatory signals to cardiomyocytes without themselves executing the death program attributed to the myocardium. The S100A9 report describes macrophage-associated signaling, STAT3 perturbation, and altered cardiomyocyte mitochondrial dynamics and mitophagy in its abstract. Because this record was not included in the focused full-text reassessment, it is used to nominate a communication route, not to certify all lineage-specific or rescue criteria. Macrophage-derived inflammation, macrophage death, and recipient cardiomyocyte death remain separate claims (68, 69, 76).

Fibroblast activation is not a death readout. Cardiomyocyte-derived small extracellular vesicles altered fibroblast activation in diabetic models, and miR-194-3p manipulation was linked to TGF*β*R2-dependent responses. These experiments support a cargo-associated communication mechanism. They do not demonstrate ferroptotic or pyroptotic execution in fibroblasts, nor do they establish core MQC as the necessary donor-cell mechanism (19, 78, 79).

### 5.3 Extracellular vesicles, mtDNA cargo, and organelle transfer

The IL-37 study provides a mitochondrial-associated communication example. Cardiomyocyte interventions altered vesicular mtDNA abundance and the profibrotic response of recipient fibroblasts; vesicle-transfer and signaling perturbations tested parts of this route. However, vesicles contain multiple cargos, and the reported experiments do not cleanly isolate mtDNA as the only necessary transferred mediator. Fibroblast inflammatory or profibrotic signaling also does not identify pyroptotic death. The evidence therefore supports a vesicle-associated communication route with a mitochondrial link, not a demonstrated ferroptosis-to-pyroptosis relay (14, 19, 80).

### 5.4 From local injury to heart-failure remodeling

Multicellular propagation may link local death to ventricular stiffness and clinical dysfunction. Endothelial impairment, immune recruitment and fibroblast activation can convert scattered cardiomyocyte stress into diffuse matrix remodeling and impaired relaxation and thereby contribute to HFpEF. Continued myocyte loss and energetic failure may later contribute to systolic dysfunction. This remains a working model because longitudinal human data connecting cellular states to phenotype transitions are limited (19, 23, 81).

Sex, age, ethnicity, obesity, kidney disease and heart-failure phenotype may alter both MQC biology and treatment response. The ARISE-HF sex analysis illustrates baseline phenotypic differences without establishing a sex-specific MQC mechanism or a definitive treatment interaction. Precision phenotyping should report representation, test interactions prospectively and avoid defining one universal DbCM endotype. Matching therapies to patients will require combined clinical, imaging and mechanism-informed variables (81–83).

## 6 Human relevance and therapeutic interpretation

Human relevance, mechanistic validation, target engagement, and clinical efficacy answer different questions. Evidence at one level cannot substitute for another (Table 5).

**Table 5:** Distinct translational claims and experimental therapeutic categories.

Evidence or strategy	What a positive finding supports	What it does not establish
Human relevance: tissue, cohorts, imaging	Association with disease phenotype or prognosis	Death mechanism or causal mediation; confounding remains
Human mechanistic model: iPSC cells or EHT	A tested mechanism within that model, if perturbation and relevant readouts are included	Patient efficacy, chronic disease fidelity, or target engagement merely from human origin
Target engagement	Intervention-linked effect on the intended molecular process in relevant tissue or a validated proxy	That the target mediates functional benefit
Clinical efficacy	Benefit on prespecified patient outcomes in the trial population	An MQC or RCD mechanism without a mediation test
Experimental MQC interventions	Potential modulation of dynamics, turnover, replacement, or proteostasis (12, 46–48)	A class-wide benefit; overactivation and energetic depletion remain concerns
Experimental ferroptosis interventions	Lipid-peroxide-sensitive rescue when measured; ferritin or defense-system mechanisms require separate tests (13, 16, 92, 93)	Specificity from antioxidant protection alone or clinical readiness
Experimental inflammatory/pyroptosis interventions	Effects on the measured node: sensing, cytokines, ASC, or gasdermins (17, 94, 95)	Prevention of lytic death from cytokine suppression alone; systemic immune safety

### 6.1 Model triangulation across cells, animals, and human cardiac systems

Common DbCM models reproduce different components of the disease. High-glucose cell culture lacks systemic metabolism and multicellular architecture; streptozotocin models emphasize insulin deficiency; db/db and diet-induced models capture obesity and insulin resistance but differ from long-duration human disease. Sex, age and comorbidity representation is often narrow. Model selection should follow the disease stage and causal question rather than convenience, and conclusions should be restricted to the construct actually reproduced (3, 4, 22).

Human engineered heart tissue provides controlled tests of human-cell responses to metabolic stress and treatment. Such experiments can support a mechanism in that model when the relevant perturbations and measurements are included; human origin alone does not establish target engagement or disease causality. Patient myocardium and clinical cohorts provide complementary disease context, but chronic comorbidity and systemic drug effects remain difficult to separate (4, 22, 49).

### 6.2 Biomarkers and imaging require explicit mechanistic roles

Each biomarker needs a defined role: susceptibility marker, mechanism marker, pharmacodynamic target-engagement marker or outcome-predictive marker. Circulating mtDNA, lipid-peroxidation products, and inflammatory cytokines may occupy more than one role. None can be assumed to identify myocardial ferroptosis, pyroptosis, or MQC failure without tissue-based or intervention-linked validation. The distinction avoids conflating biological plausibility with clinical qualification.

Circulating mtDNA, extracellular-vesicle cargo, lipid-peroxidation products, iron indices, and inflammatory cytokines are candidate correlates of injury. In the evidence assessed here, they are not validated as specific measures of myocardial core-MQC failure or a particular death executor. Associations may reflect kidney function, systemic inflammation, metabolic control, or noncardiac tissue injury. A treatment-related change in such a marker is not automatically myocardial target engagement (84–86).

CMR, echocardiographic strain, exercise capacity and circulating biomarkers capture complementary dimensions of DbCM. Imaging can identify steatosis, fibrosis, perfusion abnormalities and subtle dysfunction, but it rarely identifies a molecular death mechanism. Multimodal studies need to align imaging changes with repeated biomarkers and clinical outcomes. Longitudinal trajectories are more informative than cross-sectional thresholds because they allow investigators to test whether a mechanism becomes active before structural or functional deterioration (87–89).

### 6.3 Metabolic load and cardiometabolic therapies

Metabolic interventions can reduce upstream stress, but protection in prevention models does not establish reversal of established remodeling. Systemic improvements in glucose, lipids, weight, renal function, or hemodynamics may account for cardiac benefit without a direct MQC mechanism. This alternative is especially important for global genetic models and pleiotropic drugs (32, 34, 38).

Established cardiometabolic therapies and experimental MQC-directed interventions should not be placed at the same evidential level. Reports linking SGLT2 inhibitors or other drugs to mitochondrial effects provide candidate mechanisms; they do not show that MQC mediates clinical benefit. Clinical efficacy in a specified trial population must be distinguished from experimental mitochondrial effects and from a DbCM-specific death mechanism (47, 90, 91).

### 6.4 Experimental MQC and RCD targeting

Experimental interventions targeting mitochondrial turnover, iron/lipid-peroxide handling, or innate immune signaling remain mechanistically heterogeneous. Table 5 separates their intended targets from the evidence needed to show engagement, death-specific rescue, and patient benefit. The focused audit illustrates the central limitation: a protective intervention can improve mitochondrial and injury readouts without establishing either core-MQC mediation or a specific death executor. These candidates should therefore be described as preclinical strategies, with exposure, off-target effects, disease stage, and safety unresolved (16, 17, 46–48, 92–95).

### 6.5 Negative evidence, endotype selection, and rational combinations

A translational program needs prespecified criteria for what would count as mechanistic failure. Absence of myocardial or validated proxy target engagement argues against the intervention, whereas target engagement without functional benefit challenges the causal importance or therapeutic timing of the target. A negative clinical endpoint despite improved circulating markers should not be reclassified as success. With these decision rules, null results remain informative.

Negative trials constrain mechanistic claims. The ARISE-HF trial of aldose-reductase inhibition did not improve its primary exercise endpoint despite a plausible metabolic rationale, which illustrates the gap between pathway correction and patient benefit. Future trials should incorporate pharmacodynamic target engagement, baseline endotyping, therapeutic rather than purely preventive timing, and prespecified negative criteria. Combination therapy should proceed only after single-pathway rescue, interaction and safety have been demonstrated (71, 83, 96).

## 7 Discussion: discriminating experiments and conclusions

Recent DbCM reviews have mapped ferroptosis mechanisms and candidate interventions in detail (59, 62, 63, 97). Our synthesis instead asks whether MQC can be placed causally before defined death executors and whether mixed ferroptosis-pyroptosis signatures reflect same-cell co-execution or communication between distinct cardiac cell populations. The available evidence supports the distinction as a research framework, but not as an established disease sequence.

### 7.1 What the evidence establishes

The most defensible conclusion is that separable mitochondrial maintenance processes and associated regulators can modify injury susceptibility in particular experimental settings. Stronger attribution requires the additional steps specified in Table 4. The illustrative death audit contains pharmacologically supported lipid-peroxide-sensitive injury, but no report meeting all criteria for core-MQC-mediated execution. This result should not be generalized to all 112 publications or to the entire DbCM field.

The proteostasis and vesicle studies show why the boundaries matter. Dusp15-mtHsp70 experiments probe a core maintenance mechanism without identifying ferroptosis or pyroptosis. IL-37 and vesicle-transfer studies probe communication without establishing a two-program death relay. Combining these separate findings into a single causal pathway would exceed the evidence (12, 14, 19).

The available material does not identify a single study spanning marker-to-execution attribution, ferroptosis-pyroptosis coupling, and replication of that mechanism in human disease. The absence of such an integrated study is a limit of the evidence, not evidence that coupling is impossible. The working model is consequently modular susceptibility, with same-cell coupling and cross-cell death propagation retained as competing hypotheses (8, 10, 11).

### 7.2 A research agenda that can falsify the framework

A first priority is to determine whether a defined core-MQC defect precedes the relevant death process in a specified cell type. Serial measurements and inducible intervention can distinguish initiation from secondary mitochondrial damage. A negative result after verified MQC restoration would narrow the proposed relationship rather than justify adding more nonspecific markers (4, 25, 26).

A second priority is the same-cell versus cross-cell comparison in Table 1. Spatial profiling should nominate donor and recipient populations, followed by targeted interruption of the proposed transfer route and independent identification of death in each compartment. This design would distinguish inflammatory communication from propagation of regulated death (14, 18, 19, 23).

A third priority is replication of the specified mechanism in a human-relevant system, followed separately by evidence of myocardial target engagement and clinical benefit. Concordant direction and ordering matter more than identical effect size across models. Neither human-cell origin nor an observational patient association closes the translational gap (3, 4).

Understudied structural and proteostatic processes should be pursued when they can discriminate mechanisms, not simply expand the pathway inventory. Examples include a defined cardiolipin species, a chaperone phosphosite, or vesicular cargo whose manipulation changes the predicted downstream process. Each would require a direct link to the claim being tested (12–14, 84, 85).

Combination interventions become interpretable only after the individual mechanisms are resolved. Factorial designs can test additivity, redundancy, or compensation, while target engagement without functional benefit can identify an ineffective target or treatment window. Improvement in circulating markers alone should not override a negative cardiac endpoint (38, 71, 96).

### 7.3 Limitations of the current evidence base

The source collection was selected by the authors and lacks a reproducible original search history, complete screening log, and formal risk-of-bias assessment. Selection and availability bias cannot be quantified. Full-text availability for 66 records does not imply that all 66 underwent a complete, standardized appraisal; 46 records were initially abstract-only, and the revision-stage full-text audit intentionally covers only ten illustrative reports. The seven-study death denominator is too selective for field-wide estimates. No independent duplicate assessment or inter-rater reliability statistic is available. These limitations constrain the synthesis even after its claims have been narrowed.

The primary evidence also has important limits. Many models represent short-term glucose or lipid stress, use cardiomyoblast lines or restricted rodent populations, and do not reproduce the heterogeneity of human DbCM. Static readouts, pleiotropic inhibitors, global genetic effects, and incomplete executor-specific rescue leave competing explanations unresolved. Bulk tissue can mix different cell populations, and human observations rarely combine tissue mechanism, perturbation, and longitudinal outcome. The framework is therefore a means of formulating and rejecting mechanistic claims, not a validated causal hierarchy or a basis for selecting clinical therapy.

### 7.4 Conclusions

MQC is best treated as interacting, experimentally separable maintenance processes that may alter susceptibility to particular death programs. Selected studies support components of this model, but do not establish a universal MQC hierarchy, same-cell ferroptosis-pyroptosis co-execution, or a clinically validated death pathway. The next decisive studies should connect a specified MQC process to identified execution and distinguish within-cell coupling from communication between cells before testing the corresponding human mechanism.
